# Primary Motor Cortex Excitability in Karate Athletes: A Transcranial Magnetic Stimulation Study

**DOI:** 10.3389/fphys.2017.00695

**Published:** 2017-09-12

**Authors:** Vincenzo Monda, Anna Valenzano, Fiorenzo Moscatelli, Monica Salerno, Francesco Sessa, Antonio I. Triggiani, Andrea Viggiano, Laura Capranica, Gabriella Marsala, Vincenzo De Luca, Luigi Cipolloni, Maria Ruberto, Francesco Precenzano, Marco Carotenuto, Christian Zammit, Monica Gelzo, Marcellino Monda, Giuseppe Cibelli, Giovanni Messina, Antonietta Messina

**Affiliations:** ^1^Department of Experimental Medicine, Università degli Studi della Campania “Luigi Vanvitelli” Naples, Italy; ^2^Department of Clinical and Experimental Medicine, University of Foggia Foggia, Italy; ^3^Department of Medicine and Surgery, University of Salerno Salerno, Italy; ^4^Department of Motor, Human and Health Science, University of Rome, “Foro Italico” Rome, Italy; ^5^Struttura Complessa di Farmacia, Azienda Ospedaliero-Universitaria Foggia, Italy; ^6^Department of Psychiatry, University of Toronto Toronto, ON, Canada; ^7^Department of Anatomical, Histological, Forensic and Orthopaedic Sciences, Università degli Studi di Roma La Sapienza Rome, Italy; ^8^Department of Medical-Surgical and Dental Specialties, Università degli Studi della Campania “Luigi Vanvitelli” Naples, Italy; ^9^Department of Mental Health, Physical and Preventive Medicine, Clinic of Child and Adolescent Neuropsychiatry, Università degli Studi della Campania “Luigi Vanvitelli” Naples, Italy; ^10^Anatomy Department, Faculty of Medicine and Surgery, University of Malta Msida, Malta; ^11^Department of Molecular Medicine and Medical Biotechnology, Università degli Studi di Napoli Federico II Naples, Italy

**Keywords:** neural plasticity, transcranial magnetic stimulation, cortical excitability, motor threshold, motor evoked potential

## Abstract

**Purpose:** The mechanisms involved in the coordination of muscle activity are not completely known: to investigate adaptive changes in human motor cortex Transcranial magnetic stimulation (TMS) was often used. The sport models are frequently used to study how the training may affect the corticospinal system excitability: Karate represents a valuable sport model for this kind of investigations for its high levels of coordination required to athletes. This study was aimed at examining possible changes in the resting motor threshold (rMT) and in the corticospinal response in karate athletes, and at determining whether athletes are characterized by a specific value of rMT.

**Methods:** We recruited 25 right-handed young karate athletes and 25 matched non-athletes. TMS was applied to primary motor cortex (M1). Motor evoked potential (MEP) were recorded by two electrodes placed above the first dorsal interosseous (FDI) muscle. We considered MEP latencies and amplitudes at rMT, 110% of rMT, and 120% of rMT.

**Results:** The two groups were similar for age (*p* > 0.05), height (*p* > 0.05) and body mass (*p* > 0.05). The TMS had a 70-mm figure-of-eight coil and a maximum output of 2.2 T, placed over the left motor cortex. During the stimulation, a mechanical arm kept the coil tangential to the scalp, with the handle at 45° respect to the midline. The SofTaxic navigator system (E.M.S. Italy, www.emsmedical.net) was used in order to correctly identifying and repeating the stimulation for every subject. Compared to non-athletes, athletes showed a lower resting motor threshold (*p* < 0.001). Furthermore, athletes had a lower MEP latency (*p* < 0.001) and a higher MEP amplitude (*p* < 0.001) compared to non-athletes. Moreover, a ROC curve for rMT was found significant (area: 0.907; sensitivity 84%, specificity 76%).

**Conclusions:** As the main finding, the present study showed significant differences in cortical excitability between athletes and non-athletes. The training can improve cortical excitability inducing athletes' modifications, as demonstrated in rMT and MEP values. These finding support the hypothesis that the sport practice determines specific brain organizations in relationship with the sport challenges.

## Introduction

Motor training and professional experience lead to great changes in the brain (Penhune and Steele, 2012; Hardwick et al., 2013; Dai et al., 2016). As previously described in many electrophysiological studies, activation patterns of neurons in different brain areas are related to various cortical networks after long-term training (Fourkas et al., 2008; Wei and Luo, 2010; Schlaffke et al., 2014). Observing action of dancing with the mirror systems, it was described that the trained athletes showed greater bilateral activations in motor-related cortical areas (Calvo-Merino et al., 2005). For investigating various aspects of human neurophysiology, with a focus on corticospinal function, single-pulse transcranial magnetic stimulation (TMS) represent a useful tool (Fitzgerald et al., 2006). In classic TMS experiments, motor evoked potentials (MEPs) from muscles activities are recorded thanks to electromyographic (EMG) electrodes after stimulation of the primary motor cortex (M1). The threshold intensity can be defined as the intensity that evokes MEPs of a given amplitude during consecutive trials in a muscle. The intensity of TMS can be set to a percentage of this threshold intensity, but, since it is different for every subject, a measurement of this threshold has to be done (Wassermann, 1998).

TMS is well appreciated in the characterization of the neuromuscular responses to cortical stimulation. It has been used to study pathological conditions (Rothwell, 2011), mechanisms of fatigue in small, isolated muscle groups (Taylor and Gandevia, 2008), corticospinal contributions to human gait (Goodall et al., 2014), and acute neural adaptations following strength training (Carroll et al., 2001; Gruber et al., 2009; Tibullo et al., 2013; Moscatelli et al., 2016c).

To investigate adaptive changes in human motor cortex TMS and neuroimaging techniques were largely used (Pascual-Leone et al., 1995; Missitzi et al., 2011; Chieffi et al., 2014; Viggiano et al., 2016), contributing to the understanding of how brain networks organize the optimal motor programs which coordinate muscle activity involved in several tasks of motor learning (Nielsen and Cohen, 2008). In TMS studies, motor cortex excitability has become fundamental for the assessment of the MEP of peripheral muscles (Lee et al., 2010). The sports activity can be repetitive or situational: in the repetitive model, such as ballistic movements of the fingers, the motor cortex adaptations showed similarities with motor learning processes. The repetitive training is associated with an immediate increase in MEP response, and it was shown are in cross-sectional studies that there are similar changes among subjects with different degrees of motor skill (Selvanayagam et al., 2011).

Karate is one of the most popular martial arts practiced worldwide. Karate contains two main practices (i.e., *kata* and *kumite*). *Kata* represents a demonstrating pre-arranged form of methods of defense, attack, and counter attack. In this training, a performance of set sequences of basic techniques in a fight with an imaginary opponent is performed. *Kumite* represents the process of sparring utilizing karate skills performed by two opponents. *Kumite* requires the karateka, a rapid choice of the adequate action pattern to defense and attack (Tan, 2004; Tabben et al., 2013; Chaabne et al., 2015). High technical skills (i.e., kick and punch) are required in fight (i.e., kumite) and high levels of precision and velocity are needed to execute the right movements for attack and defense (Kim and Petrakis, 1998; Mori et al., 2002; Chieffi et al., 2014; Viggiano et al., 2014; Messina et al., 2015). In addition, cognitive abilities and efficient attentional processes influence the technical performance in karate allowing more time for the organization of motor behavior; furthermore, quick and correct responses are ensured to visuospatial stimuli (Andreato et al., 2013; Bridge et al., 2014). Karate, with its high levels of coordination required, was found ideal to investigate the effects of training on athletes' corticospinal system excitability (Moscatelli et al., 2015). Moreover, it was found that the motor control differences between expert and novice karate athletes are strictly related to white matter structure in M1 and in the cerebellum (Roberts et al., 2013).

In general, karate athletes tend to show better physical fitness indices compared with their control such as higher muscular endurance and flexibility. Moreover, about the DTI studies data athletic groups show significantly lower fractional anisotropy (FA) and marginally higher mean diffusivity (MD) values in the globus pallidus internal segment (GPi) compared with control group. These findings suggest that professional sport is associated with changes in white matter integrity in specific regions of the basal ganglia (Chang et al., 2015).

The specific behavioral demands of the training experience can highly influence the motor cortex reorganization. Comparing less proficient players and non-playing controls with highly skilled racket players were found a larger hand motor representation together with higher MEP amplitudes (Pearce et al., 2000; Barone et al., 2017). Similar results were reported analyzing highly skilled volleyball players vs. runners, showing, the former, higher overlapped representations of carpi radials muscles and medial deltoid (Tyc et al., 2005). Furthermore, recent studies showed that there is a correlation between motor coordination and cortical excitability (Moscatelli et al., 2016a), and between muscle fatigue and cortical excitability (Moscatelli et al., 2016b,c). The aim of this study was to verify whether Karate athletes have a different resting motor threshold (rMT) and corticospinal response (MEP) compared to controls. Moreover, using Receiver Operating Characteristic (ROC) curve, we investigated if athletes are characterized by a specific value of rMT.

## Materials and methods

### Subjects

We enrolled 25 karate athletes and 25 matched non-athletes were recruited (the anthropometric measurement in Table 1). All the athletes were males, and right-handed, as assessed by Edinburgh Handedness Inventory (Oldfield, 1971). All procedures were conformed to the directives of the Declaration of Helsinki and were approved by the Institutional Ethical Committee of the University of Foggia. The athletes were Caucasian karate black belts. They competed at national and international levels; they trained at least five 2-h sessions every week during the previous 5 years. The control group was composed of people did not engage in any competitive or amateur sport. Two days before the recordings, all subjects did not perform physical activity. An informed consent was subscribed by all participants: all possible risks correlated with the experimental model were illustrated. Moreover, a medical assessment confirmed the absence of psychoactive or vasoactive medication assumption, risk factors, and any kind of contraindications (Rossini et al., 2015). All subjects sign an informed consent before the experimental task.

**Table 1:** Anthropometric characteristics of the experimental groups.

**Parameter**	**Karate athletes**	**Non-athletes**	***p*-value**
Age (year)	24.9 ± 4.9	26.2 ± 4.5	>0.05
Height (cm)	176.1 ± 3.9	176.3 ± 7.2	>0.05
Body mass (kg)	78.1 ± 11.4	80.7 ± 10.4	>0.05

*Values are expressed as mean ± standard deviation*.

### Methodology

During the experimentation, the testing was done under well-established standard conditions: for example, all measurements were carried out between 2:00 and 4:00 p.m.; furthermore, the environmental conditions were the same for each test (comfortable armchair, quiet room, elbow positioned at 90° flexion). The TMS had a 70-mm figure-of-eight coil and a maximum output of 2.2 T (Magstim Rapid^2^, The Magstim Company Ltd, UK). The coil was placed over the left motor cortex. During the stimulation, a mechanical arm kept the coil tangential to the scalp, with the handle at 45° respect to the midline. We used the SofTaxic navigator system (E.M.S. Italy, www.emsmedical.net) to correctly identify and repeat the stimulation for every subject. Individual resting motor threshold (rMT) was determined by stimulating the left primary motor cortex, following a standardized procedure (Rossini et al., 1994). Evoked muscle responses were recorded using the BioPack MP150 (BIOPAC Systems, Inc., CA, USA), through two surface electrodes (1 cm of diameter) placed over the left index finger: the active electrode was placed over the first dorsal interosseous (FDI) muscle; the reference electrode was placed over the associated joint or tendon; the ground electrode was placed over the dorsal part of the forearm. The recorded EMG signals were pre-processed and analyzed offline, using the Acknowledge software, version 4.1 (BIOPAC Systems, Inc., CA, USA) with a high pass filter (frequency cutoff: 10 Hz).

The signal analysis was focused to extract three neurophysiological parameters: rMT, MEP latency, and MEP amplitude. MEP is the electrical potential recorded from a muscle after a direct stimulation of the motor cortex if its intensity is higher than the rMT. This threshold can be defined as the lowest intensity needed to have the 50% probability of eliciting a 50 μV MEP when the muscle is completely relaxed. This value varies among the population and different muscles. rMT was measured at rest, expressed as a percentage of the maximal stimulator output.

Therefore, to ensure the same relative intensity of stimulation for every subject, the intensity of stimulation was set at 110–120% of the rMT, the “basic unit of dosing” (Borckardt et al., 2006). MEP latencies (i.e., the velocity at which the neural signal is propagated from the motor cortex to the muscle) and amplitudes (i.e., the magnitude of corticospinal excitability) at rMT, 110% of rMT (110%rMT), and 120% of rMT (120%rMT) were measured by means of the recordings.

MEP latency was the time between the trigger itself (onset of the square wave) and the start of muscle response. For rMT condition, five responses were averaged. For 110% rMT and 120% rMT conditions, we averaged ten successive responses.

### Statistical analysis

R Project for Statistical Computing software (version 3.1.0) was used for the statistical analysis. Significance was set at *p* < 0.05. The normality of distribution of variables was checked using the Shapiro–Wilk test. The differences between groups for rMT were evaluated using *t*-test. The differences between groups for MEP latency and amplitude was tested using repeated measure ANOVA, followed by Tukey's HSD (honest significant difference) as *post-hoc* comparisons. The classification rate was computed by the analysis of the ROC curve (DeLong et al., 1988).

## Results

During the experiments, no adverse effects or discomfort were reported.

**Figure 1:**
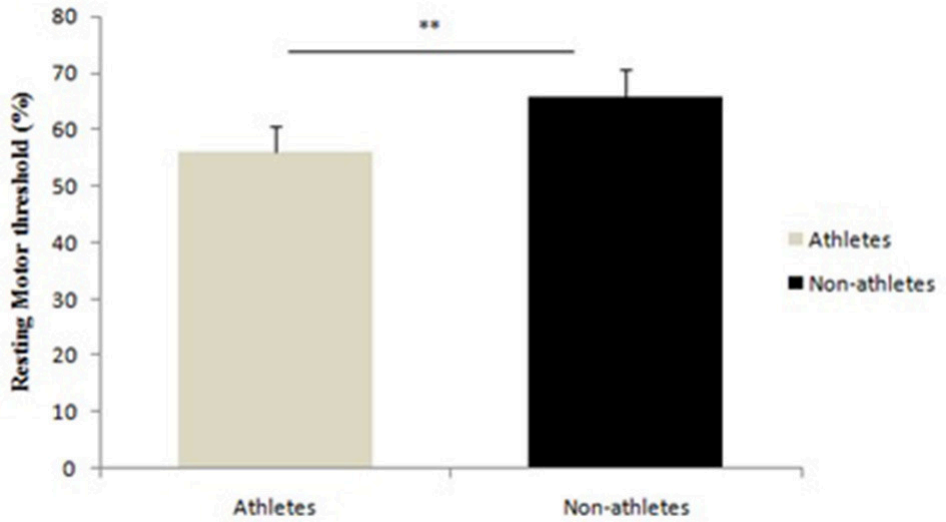
Differences between athletes and non-athletes in resting motor threshold, ^**^p < 0.001.

Compared to controls (64.9 ± 4.6% of maximum output), karate athletes had a lower (*p* < 0.01) rMT (57.1 ± 4.2% of maximum output). The result is shown in Figure 1.

Furthermore, in karate athletes a shorter MEP latencies were observed [*F*_(5, 144)_ = 26.13; *p* < 0.0001] respect to the control group. *Post-hoc* analysis showed differences between groups for rMT (*p* < 0.01), for 110% rMT (*p* < 0.01), and for 120%rMT (*p* < 0.01).

Moreover, in karate athletes a higher MEP amplitudes were observed [*F*_(3, 92)_ = 217.7; *p* < 0.0001] respect to the control group. *Post-hoc* analysis showed differences between groups for 110% rMT (*p* < 0.01; Table 2).

**Table 2:** Motor evoked potential latency and amplitude results.

**Inyensity of stimulation**	**Parameters**	**Athletes**	**Non-athletes**	***p*-value**	**Δ%**
rMTintensity	Latency (ms)	22.16 ± 1.7	24.75 ± 1.1	<0.0001	10.46
	Amplitude (mV)	90.11 ± 10.1	59.98 ± 13.4	<0.001	−33.44
110% of rMT	Latency (ms)	21.37 ± 2.0	24.2 ± 2.3	<0.0001	11.69
	Amplitude (mV)	236.77 ± 29.1	137.16 ± 33.2	<0.01	−42.07
120% of rMT	Latency (ms)	20.48 ± 1.8	23.42 ± 1.4	<0.0001	12.55
	Amplitude (mV)	249.12 ± 45.9	258.12 ± 65.9	>0.05	3.61

*Values are expressed in mean ± standard deviation. rMT, resting motor threshold*.

**Figure 2:**
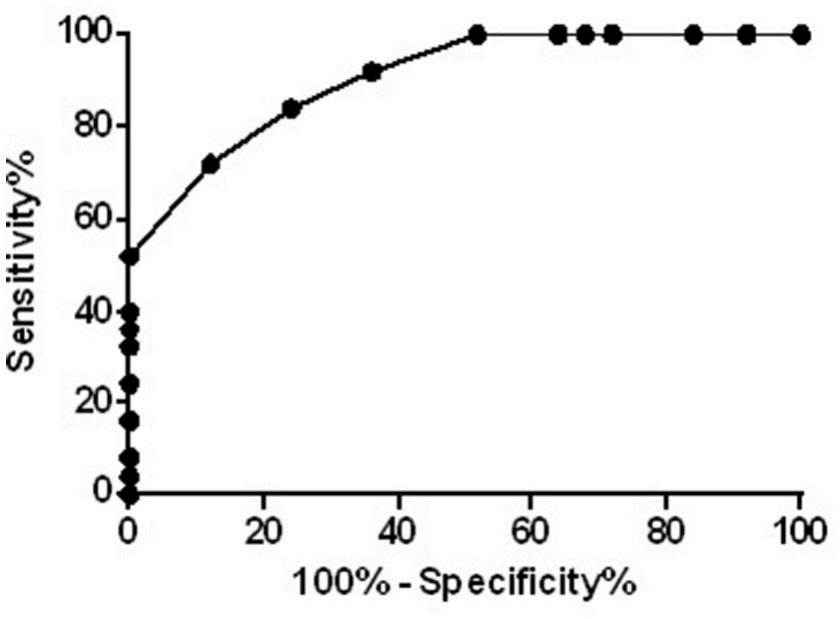
ROC curve of the resting motor threshold considering the whole of population, ^**^p < 0.001.

In Figure 2 is shown the ROC analysis of the sensitivity and specificity of the resting motor threshold considering the whole of karate athletes population after TMS. P values were from the comparisons of the ROC AUC (area under curve) of specificity vs. ROC AUC of sensitivity, ^**^p < 0.001 (sensitivity 84%, specificity 76%; accuracy 80%; area 0.907).

## Discussion

As the main finding, this study showed that athletes had significant differences in cortical excitability compared to non-athletes and are characterized by a specific value of rMT as showed by ROC curve. Moreover, MEP response showed that athletes had lower rMT (%), shorter MEP latency (ms), and higher MEP amplitude (mV) compared to non-athletes. These findings support the hypothesis that training results in specific changes in brain cortex connectivity to meet specific sport challenges.

People faced continuously the opportunity to learn and perform their own motor skills, being motor control an essential part of life. Motor skills how well neural plasticity and cortical reorganization influence can be acquired and learned. The cortical representations are increased with the use and repetition through motor practice. According to literature (Nielsen and Cohen, 2008), different M1 excitability reflects the neural plasticity substrate responsible for the acquisition and maintenance of specific motor skills. Similarly, decrease in rMT was seen in subjects trained to produce skilled finger movements (piano playing). Furthermore, compared to controls, subjects trained on skilled ankle (Perez et al., 2004) and tongue tasks (Svensson et al., 2006) showed increased MEP amplitude and enhanced movement-representation areas.

The effect of motor learning was investigated with neuroanatomical, imaging, neuromagnetic, and electrophysiological studies. These studies revealed a higher activation in the contralateral primary sensory cortex, the dorsal premotor cortex, the supplementary motor area, the secondary sensory cortex bilaterally, and the posterior parietal cortex (Golaszewski et al., 2004; Wu et al., 2005; Manto et al., 2006; Messina et al., 2014, 2016; Moscatelli et al., 2016b). Moreover, it was established a ready neuromuscular adaptation due to resistance exercise training (Hortobágyi et al., 1996; Aagaard et al., 2001, 2002; Li Volti et al., 2011). Thanks to some direct measurements (e.g. histological staining, CT, and fMRI), many morphological changes can be determined although they cannot completely explain the acute responses to motor tasks and resistance exercise. Those are reasonable neural-derived during the early phases of training (Hortobágyi et al., 1996; Aagaard et al., 2001, 2002).

Changes in rMT and MEP values in athletes might be due to the plasticity of the motor cortex related to sport practice, expression of an increased cortical excitability, which might be in turn attributed to plasticity of the motor cortex. It is commonly accepted that an increased MEP amplitude is an index of the plasticity response due to motor skill learning (Francavilla et al., 2008; Cirillo et al., 2011). In fact, MEP amplitude of the target muscles increased after a simple motor-training intervention. This was considered a reflection of the use-dependent plasticity, via long-term potentiation-like mechanisms in brain cortex (Goodall et al., 2014; Grospretre et al., 2015; Pomara et al., 2016). In other studies was described that visuomotor tracking tasks in young subjects provokes the increase in MEP amplitude (Perez et al., 2004; Todd et al., 2009; Viggiano et al., 2014); such complex tasks require a specific high attentional focus, to reach higher levels of performance, and rely on processes within cortical circuits (McNevin et al., 2000). The continuous practice of movements, like the ballistic movements of the fingers, lead to adaptations within the motor cortex similar to motor learning related adaptations.

The performance improvement is usually accompanied by an immediate increase in MEP response (Missitzi et al., 2011). Another result showed how a single session of strength training induces changes in corticospinal excitability similar to those obtained after the early steps of a ballistic motor learning task (Selvanayagam et al., 2011). The results of this study have shown that also a single session of strength training brings to neural changes similar to those related to the ballistic motor learning. Several studies described the reorganization of functional representation in the sensorimotor cortex (Alagona et al., 2009; Selvanayagam et al., 2011). Evidence for the effects of motor training and skill acquisition on the functional representation of the hand has come from studies on the monkeys and in subjects learning a one-handed five-finger exercise on the piano (Pascual-Leone et al., 1995). TMS-based experiments studied the corticospinal excitability during motor training. Most of the results showed that the changes due to the training are related to a MEP amplitude increase in the target muscles. Furthermore, it was seen that even a few-minutes training may induce changes in cortical excitability, changes that last several minutes (Gallasch et al., 2009). Unfortunately, there are no studies investigating long-term training effects. On the other hand, a lot of TMS studies reported enhancement of MEP amplitude during motor imagery (MI) for upper and lower limb movements (Buccino et al., 2001; Muellbacher et al., 2002; Grospretre et al., 2015; Moscatelli et al., 2016c), reflecting an increase in the cortical-spinal excitability. As an example, MEP threshold resulted lower during MI, maybe due to a higher neuronal response to the magnetic stimulation (Muellbacher et al., 2002) and a lower intracortical inhibition. MI of hand was also seen bringing to a spread of those brain areas which control the opponents pollicis muscles and the abductor digiti minimi (Viggiano et al., 2016). This experimental evidence showed that cortical cell responsiveness might increase during MI, allowing the recruitment of a larger number of neural structures when discharging a magnetic pulse.

The differences between athlete and non-athletes in this study could be attributed to a better training-related cortical connectivity in karate athletes. Of note, adult brains can modify their organization throughout physiological mechanisms (i.e., the repetitions of a simple movement; Pearce et al., 2000). If the environment changes, the brain plasticity enables the nervous system to ensure the muscles activation to serve the behavioral goal; more recently it was reported that the brain plasticity is determined by a genetic component (Missitzi et al., 2011; Salomone et al., 2013; Valenzano et al., 2016).

To our knowledge, this study is the first investigation regarding the specific effect of long term training in the brain. In this study emerged that athletes show higher cortical excitability of M1 and are characterized by a specific value of rMT. However, future studies might be focused on the explanation of the nature of the emerging differences in cortical excitability between trained and not trained subjects.

## Ethics statement

All procedures are conformed to the directives of the Declaration of Helsinki. The Institutional Ethics Committee of the University of Foggia approved the study. All subjects gave their written informed consent before participation.

## Author contributions

VM, AV, FM, AM: Conceived the study, participated in its design. MS, FS, AT, LC, GaMa, VD, LC, MR, MG, FP: Contributed to the conception and design. AV, VM, FM, GM, AM wrote manuscript. MC, CZ, AnVi, MM, GC, GM drafted the article and revised it critically for important intellectual content; GM, AM: Final approval of the version to be published. All authors read and approved the final manuscript.

### Conflict of interest statement

The authors declare that the research was conducted in the absence of any commercial or financial relationships that could be construed as a potential conflict of interest.
